# Design-Dependent Myopia Control in Orthokeratology: Spherical Versus Aspherical Back Optic Zone Profiles

**DOI:** 10.3390/bioengineering13040414

**Published:** 2026-04-01

**Authors:** Wen-Pin Lin, Huibin Lv, Lo-Yu Wu, Richard Wu, Xueli Li, Ahmed Abass

**Affiliations:** 1 Department of Optometry, Mackay Medical University, New Taipei 252, Taiwan; 2 Research and Development Centre, Brighten Optix Co., Taipei 111, Taiwan; 3 Department of Ophthalmology, Peking University Third Hospital, Beijing 100191, China; 4 College of Optometry, Pacific University, Forest Grove, OR 97116, USA; 5 Beijing Bright Care Eye Clinic Ltd., Beijing 100021, China; 6 Department of Materials, Design and Manufacturing Engineering, School of Engineering, University of Liverpool, Liverpool L69 7ZX, UK

**Keywords:** eye, orthokeratology, myopia management, myopia control, aspherical geometry, decentration, axial length

## Abstract

**Background**: This study examined spherical and aspherical orthokeratology (Ortho-K) lens designs for myopia control and corneal optical stability over the course of a year. **Methods**: This retrospective analysis used data from a previously conducted two-centre, single-blind, randomised contralateral-eye clinical study, in which 48 children aged 8 to 15 years wore a spherical Ortho-K lens in one eye and an aspherical lens in the other. Measurements included axial length (AL), best-corrected visual acuity (BCVA), lens decentration, corneal power, and higher-order aberrations over 12 months. Corneal topography was analysed using customised MATLAB code, Zernike fitting and paired inter-eye differences were evaluated with the Wilcoxon signed-rank test. **Results**: Both lenses exhibited typical Ortho-K reshaping patterns, with central flattening and mid-peripheral steepening. The aspherical lens resulted in slower AL elongation than the spherical lens (*p* < 0.01). It also produced smaller, more stable treatment zones and less variability in higher-order aberrations. Significant differences between the designs were found for vertical coma (*p* = 0.006), spherical aberration (*p* = 0.002), and vertical tilt (*p* = 0.02). Lens decentration also differed significantly (*p* < 0.01). **Conclusions**: Over 12 months, the aspherical Ortho-K lens demonstrated superior myopia control and more stable corneal optics than the spherical lens.

## 1. Introduction

Myopia, or near-sightedness, has become a pressing global public health concern [1,2,3], affecting increasing numbers of children and young adults worldwide and escalating the risk of sight complications such as retinal detachment, glaucoma, and myopic maculopathy [4]. Alongside strategies like increased outdoor activity and low-dose atropine eye drops [5,6], optical interventions, most notably orthokeratology (Ortho-K), have gained traction in efforts to slow myopic progression [7].

Ortho-K employs specially designed rigid gas-permeable (RGP) contact lenses worn overnight to temporarily reshape the corneal surface, flattening the central cornea to correct myopia during waking hours [8]. This reverse-geometry design induces controlled epithelial thinning centrally, with compensatory thickening in the mid-periphery, creating a treatment zone that maintains daytime clarity while imposing relative peripheral myopic defocus, a key signal believed to slow axial elongation in growing eyes.

The literature supports the efficacy of Ortho-K for myopia control. Meta-analyses found significant reductions in axial elongation and refractive progression in school-aged children wearing Ortho-K lenses, with mean decreases of approximately 0.53 mm in axial length (AL) and 3.22 dioptres (D) after two years [9,10]. Longitudinal clinical studies, including a multi-year cohort from Peking University, have confirmed sustained slowing of axial growth and good safety and compliance in paediatric populations [11]. Typically, reductions in axial elongation of 30–50% are observed compared to controls fitted with spectacles or soft lenses [12].

Although the precise mechanism remains under investigation, the prevailing theory suggests that induced peripheral myopic defocus alters retinal signalling, reducing the stimulus for axial growth. With its non-invasive, reversible nature and demonstrated effectiveness, Ortho-K has emerged as a valuable myopia-control tool in clinical practice, especially for children at risk of rapid progression [13].

Beyond the general efficacy of Ortho-K, increasing attention has been given to the role of specific lens design parameters in shaping myopia-control outcomes. Previous studies have shown that treatment-zone size, lens decentration, and the resulting redistribution of corneal power can all influence the optical profile generated on the eye and, in turn, axial growth [14,15,16]. In particular, smaller and more concentrated treatment zones have been associated with slower axial elongation, while lens decentration may modify both the corneal refractive pattern and treatment effect [14,16]. More recently, aspheric Ortho-K designs have been reported to alter corneal topography, peripheral refraction, and myopia-control efficacy differently from spherical designs, supporting the view that relatively subtle back optic zone (BOZ) modifications may have clinically meaningful effects [17,18]. Unlike conventional spherical designs and previously reported fully aspherical optic-zone profiles, the lens evaluated in this study incorporates a hybrid BOZ geometry in which the central zone remains spherical. At the same time, a spline-based aspheric contour is confined to the outer one-third of the BOZ. This design was intended to smooth the transition between zones, provide a more continuous alignment profile, and subtly alter reverse-curve behaviour, thereby promoting more stable corneal reshaping without compromising central optical quality. Such reshaping may also yield a smaller, more stable mid-peripheral steepening pattern, thereby supporting a more consistent peripheral myopic-defocus signal.

The authors, therefore, hypothesised that, compared with a conventional spherical design, this hybrid aspherical BOZ profile would generate a smaller and more stable treatment zone, reduce variability in corneal optics, and slow axial elongation more effectively over 12 months. To isolate the effect of lens geometry as cleanly as possible, the original clinical study was designed as a randomised contralateral-eye trial, allowing each participant to serve as their own control and thereby reducing between-subject variability. However, inter-eye correlation and binocular adaptation effects cannot be completely excluded.

Against this background, this study explores how spherical (vision-shaping treatment, VST) and aspherical BOZ geometries differ in their effects on axial growth, corneal optics, and clinical outcomes over 12 months.

## 2. Materials and Methods

### 2.1. Study Design and Participants

This retrospective analysis used data previously collected in a two-centre, single-blind, randomised contralateral-eye clinical study conducted under a shared protocol. Participants aged 8 to 15 years were recruited from the local community, and the clinical procedures relevant to the present analysis were performed at the Department of Ophthalmology, Peking University Third Hospital, Beijing, China. Inclusion criteria were: spherical refractive error between −0.75 D and −4.00 D, no or mild astigmatism (≤1.50 D with-the-rule or ≤1.00 D against-the-rule), and best-corrected monocular visual acuity (BCVA) of 1.0 decimal or better. Baseline corneal surface and eye refractive power measurements’ descriptive statistics are shown in Table 1, where the data’s central tendency, variability and most frequent values can be viewed.

All participants were first-time Ortho-K users and had not previously received any myopia-control treatment. Exclusion criteria included a history of ocular disease or surgery, abnormal intraocular pressure (IOP), keratoconus, low endothelial cell density, or an exceptionally poor lens fit during trial fitting. This retrospective analysis involved previously collected clinical data, and no new participant recruitment was undertaken. The data were derived from a two-centre clinical study conducted under a shared protocol. Ethical approval for the study protocol was granted by the Ethics Committee of North Sichuan Medical College, Sichuan, China (IRB00006761-M2024755), which served as the lead approving institution, and the clinical procedures at Peking University Third Hospital were conducted under the same approved protocol. All procedures adhered to the Declaration of Helsinki and its subsequent amendments. Written informed consent had been obtained from each participant’s guardian at the time of the original data collection, with verbal assent provided by the participants themselves. Patient confidentiality was maintained throughout data handling and analysis.

In the original clinical study, each participant’s eyes were randomly assigned to wear either a spherical or an aspherical Ortho-K lens according to a computer-generated randomisation schedule. The randomisation sequence was prepared by an independent research staff member who was not involved in participant recruitment, lens fitting, or outcome assessment. Allocation of lens design to the right or left eye was implemented using sequentially numbered, opaque, sealed envelopes, which were opened only after enrolment and completion of baseline examinations. This contralateral-eye design allowed each participant to serve as their own control. Experienced clinicians performed lens fitting, while outcome assessors were masked to lens allocation throughout follow-up.

Spherical (VST) lenses represented the conventional, symmetric Ortho-K design. By contrast, aspherical lenses had a spline profile applied to the outer one-third of the BOZ. At the same time, the central two-thirds, approximately 4 mm in diameter, remained spherical. The full BOZ spanned a 6 mm diameter optical zone on the cornea, meaning the aspheric modification was confined to the 4 to 6 mm peripheral region. This refinement subtly adjusted the height of the reverse curve (RC), the steeper zone just outside the base curve that forms the tear reservoir and drives epithelial redistribution, without changing key fitting parameters such as the flat K, overall BOZ diameter, or sagittal height.

**Figure 1:**
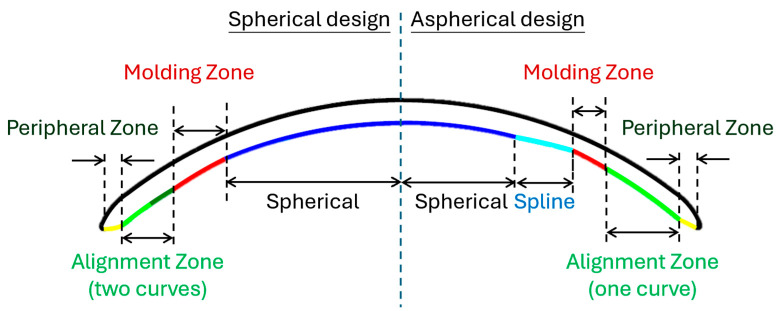
Schematic displaying the difference between the two Ortho-K lens designs used in the current study. Spherical design to the left and aspherical design to the right.

A schematic comparison of the two back-surface geometries is shown in Figure 1. The spherical design features distinct zones, including a spherical base curve, a defined moulding zone, a peripheral zone, and a two-curve alignment zone. By contrast, the aspherical design retains a central spherical optical zone but introduces a spline-based aspheric contour in the outer one-third of the back optic zone. Thus, the key structural difference lies in the transition profile: the spherical design uses discrete zone transitions, whereas the aspherical design was intended to create a smoother transition between zones and a single, continuous alignment curve, thereby enhancing the stability of lens fitting and corneal reshaping.

The spherical and aspherical Ortho-K lenses used in the study were made of high oxygen permeability (Dk) RGP materials Boston XO2 and Boston XO, respectively (Bausch + Lomb, Wilmington, MA, USA). Both designs followed standard reverse-geometry fitting protocols. Participants were instructed to wear the lenses overnight for 7 to 9 h and to follow a consistent lens care routine. Adherence was monitored through logbooks, monthly follow-ups, and caregiver reports.

In the original clinical protocol, follow-up visits were scheduled at one day, one week, one month, and then approximately monthly throughout the 12 months after lens dispensing. At each visit, several ophthalmic evaluations were performed. AL was measured using the IOLMaster 700 (Carl Zeiss Meditec, Jena, Germany), and the final value for each eye was calculated as the average of five consistent readings. Measurements were repeated when signal quality was deemed suboptimal or when obvious outliers were observed. Corneal topography was assessed using the Medmont E300 (Medmont, Nunawading, VIC, Australia) to monitor corneal reshaping and lens centration, and scans were reviewed for adequate centration, coverage, and overall image quality before analysis. IOP was measured using a non-contact tonometer NIDEK NT-530 (NIDEK, Gamagori, Japan), with the final value recorded as the average of three readings. Uncorrected and corrected visual acuity were assessed clinically, and refraction was measured using a NIDEK ARK-1 (NIDEK, Gamagori, Japan) autorefractor. Visual acuity data were analysed in decimal units, while autorefractor measurements served as the starting point for refraction.

### 2.2. Digital Signal Processing

**Figure 2:**
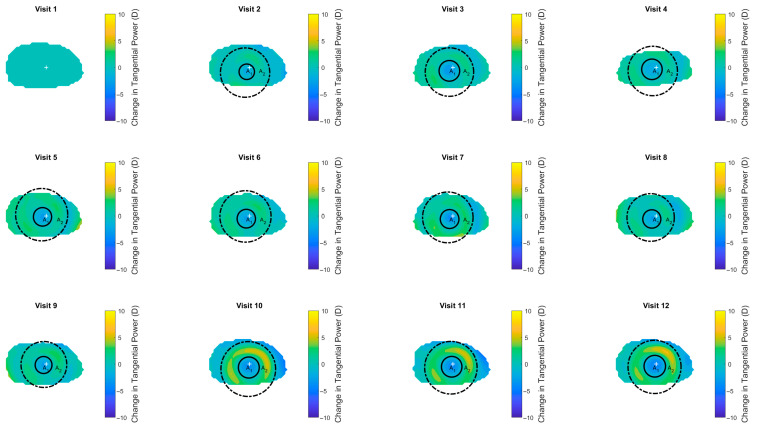
An example of an Ortho-K subject’s tangential power change between clinical monthly follow-ups. A_1_ is the central flattened zone area with average power P_1_, and A_2_ is the peripheral steepened zone area with average power P_2_. Both areas were automatically identified using a custom-built MATLAB code.

The tangential power maps were generated using a custom MATLAB 2025b script developed for this study (MathWorks, Natick, MA, USA). Raw topography data in MXF format were imported and parsed using MATLAB’s home-built reading functions to extract corneal height, curvature, and power information for each clinical visit. The code automatically identified the right or left eyes, extracted measurement dates, and sorted them chronologically. Corneal heights were reconstructed from raw data and normalised relative to the first visit. Tangential curvature and power distributions were computed through differential analysis. For each visit, the change in tangential power in dioptres was calculated relative to baseline and spatially smoothed to reduce local noise. The resulting difference values were plotted as coloured-scaled maps, displaying the evolution of corneal reshaping over time. Two key areas were automatically detected: the central flattened zone A_1_ with average tangential power P_1_, and the surrounding peripheral steepened zone A_2_ with average tangential power P_2_. These zones were identified using computerised circle-fitting algorithms applied to the tangential power difference maps [19,20], and their mean dioptric changes and spatial extents were quantified. All processing, segmentation, and visualisation were performed automatically within MATLAB to ensure consistency across visits and subjects (Figure 2).

A custom MATLAB function for Zernike fit was developed to model the corneal surface using Zernike polynomials [21]. Zernike functions form an orthogonal basis over the unit circle. They are widely used to represent optical wavefronts and corneal topography because they can describe both symmetric and asymmetric aberrations.

The accuracy of the Zernike polynomial fit to each corneal surface was evaluated using root-mean-square error (*RMSE*); lower *RMSE* values indicated a closer, more precise fit. In this context, “error” refers to the difference in surface elevation between the clinically measured corneal data and the surface reconstructed from the Zernike polynomial model. Considering the Z-axis as the direction of the AL of the eye, and for a surface grid centred on the corneal apex, the radial distance of each grid point, $\rho_{g}$, was calculated as

(1) $$\rho_{g}=\sqrt{{X_{g}}^{2}+{Y_{g}}^{2}},$$

where $X_{g}$ and $Y_{g}$ represent the Cartesian coordinates of each of the grid points in the *XY* plane. A normalised form ρ of the radius $\rho_{g}$ is required for the Zernike fit, and can be calculated as

(2) $$\rho =\frac{\rho_{g}}{\rho_{max}},$$

where $\rho_{max}$ is the maximum radius observed in the data, which in this case was set to 5 mm to ensure that the data were in Medmont’s most reliable measurement area, as peripheral measurements are less reliable. Any surface data beyond this maximum radius was disregarded in these analyses. The Zernike raw elevation $Z_{n}^{m}\left(\rho ,\varphi \right)$ is given by [21]

(3) $$Z_{n}^{m}\left(\rho ,\varphi \right)=\left\{\begin{array}{cc} R_{n}^{m}\cos\left(m\varphi \right) & m>0\\ R_{n}^{m}\sin\left(m\varphi \right) & m<0 \end{array}\right.,$$

where φ is the azimuthal angle of the coordinates $X_{g}$ and $Y_{g}$ measured parallel to the iris plane, n is the radial order of the polynomial, m is an azimuthal integer index that varies from −n to n for even (m − n) and equals 0 for odd (n − m), and $R_{n}^{m}$ is a radial polynomial, defined as

(4) $$R_{n}^{m}\left(\rho \right)=\sum_{k=0}^{\frac{n-m}{2}}\frac{{\left(-1\right)}^{k}\left(n-i\right)!\;\rho^{n-2k}}{k!\left(\frac{n\;+\;m}{2}-k\right)!\left(\frac{n\;-\;m}{2}\right)!}\;\;\;\;\;\;\;\;\;\left(0\leq \rho \leq 1\right),$$

Zernike generated surface height (raw elevation) term $Z_{n}^{m}\left(\rho ,\varphi \right)$ was fitted to the anterior corneal surfaces. The *RMSE* was calculated for every fit as

(5) $$RMSE=\sqrt{\frac{\sum_{i=1}^{q}{\left(Z_{i\;fit}-Z_{i\;surf}\right)}^{2}}{N}},$$

where $Z_{fit}$ is the Zernike fitted surface height and $Z_{surf}$ is the measured raw elevation surface height, and N is the total number of data points considered in the *RMSE* calculation.

The Zernike coefficients that represent the contribution of each polynomial term were derived using a least-squares fitting approach, where the polynomial terms were fitted to the measured corneal raw height data to minimise the overall *RMSE* difference between the reconstructed and measured surfaces.

Zernike polynomial coefficients describe different components of the eye’s optical behaviour, and a change in each term coefficient provides evidence as to how the cornea was reshaped during Ortho-K wear. The defocus-related terms reflect changes in central power. Tilt terms indicate slight shifts in the optical axis and are sensitive to lens centration. Coma components represent asymmetric distortions, with vertical and horizontal coma revealing whether the treatment zone has shifted superiorly, inferiorly, or laterally on the corneal surface. Trefoil terms describe three-lobed patterns of irregularity that arise from subtle asymmetries in the reshaped cornea.

In contrast, astigmatism terms capture meridional variations, and spherical aberration reflects the relationship between central and peripheral corneal power, which has direct implications for contrast and night-vision quality. Finally, the quadrifoil terms describe higher-order, four-point distortion patterns that highlight finer irregularities in corneal contour. Together, these Zernike coefficients offer an optical fingerprint of the remodelling induced by spherical and aspherical Ortho-K lenses.

**Figure 3:**
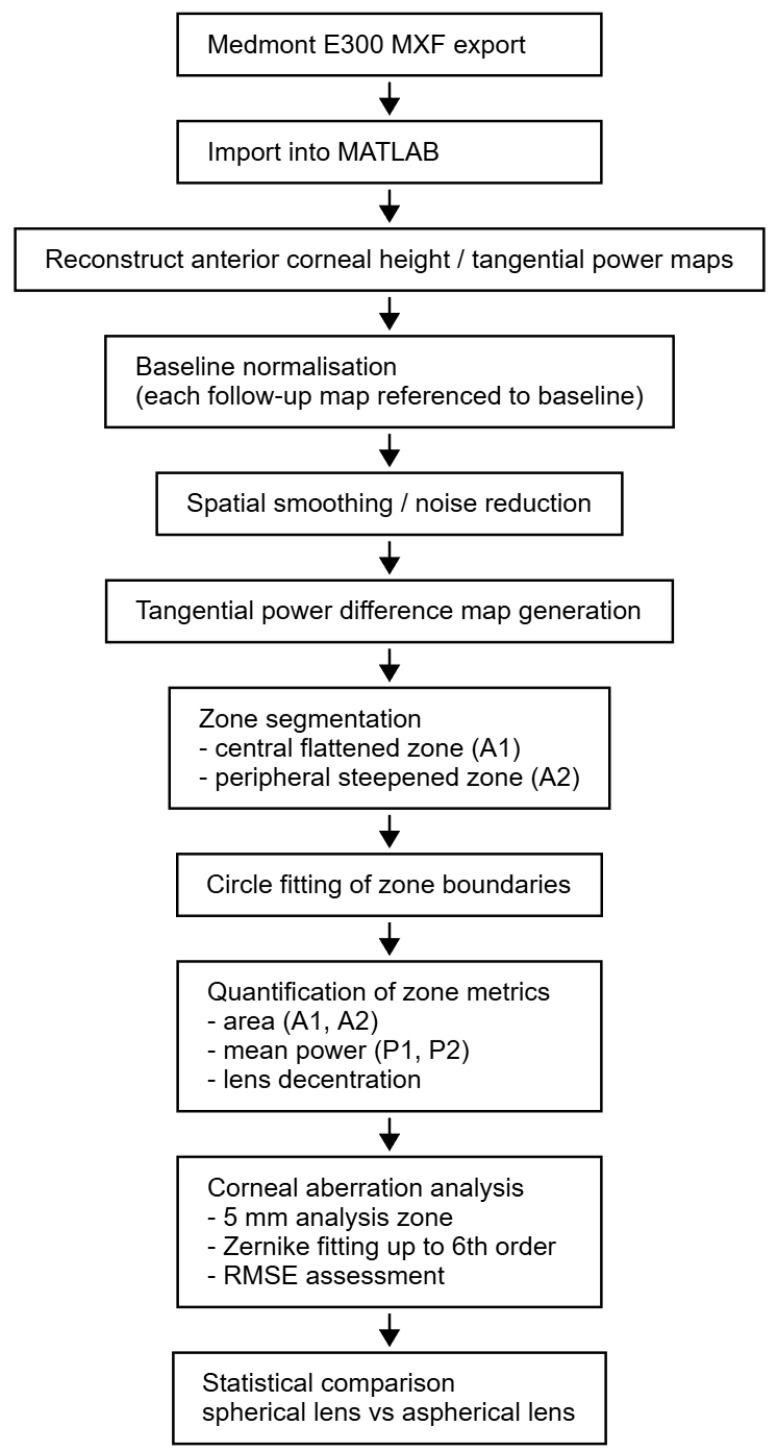
Description of the MATLAB-based processing pipeline as used in the current study.

An overview of the MATLAB-based processing pipeline and methodological details is shown in Figure 3 to enhance the reproducibility of the numerical analysis presented in the current study.

### 2.3. Statistical Analysis

A sensitivity analysis based on the completed sample of 48 paired participants indicated that, with a two-sided α of 0.05, the study had 80% power to detect a moderate paired effect size of approximately 0.41 (and 90% power for an effect size of 0.48). The study was therefore adequately powered to detect moderate inter-design differences, but smaller effects, particularly for secondary optical outcomes, may have gone undetected.

All statistical analyses were performed using MATLAB R2025b (MathWorks, USA). Because the analysed dataset arose from a randomised contralateral-eye study, the primary unit of comparison was the paired inter-eye difference within each participant rather than the individual eye treated as an independent observation. For each participant, outcomes from the spherical-lens eye were compared directly with those from the aspherical-lens eye, thereby accounting for the within-subject correlation inherent to the study design. Longitudinal measurements of AL, BCVA, corneal curvature, optical power (P_1_ and P_2_), and power-zone areas (A_1_ and A_2_) were summarised descriptively at each visit using group means and standard deviations.

Nonparametric Wilcoxon signed-rank tests were applied to paired inter-eye differences at the final 12-month visit for comparisons between spherical and aspherical Ortho-K lenses. This test was chosen because the design was paired and the sample size was modest, making a nonparametric paired approach more appropriate. Pairwise comparisons were performed for AL change, BCVA change, lens decentration, corneal tangential powers (P_1_, P_2_), and the corresponding zone areas (A_1_, A_2_). *p*-values < α were considered statistically significant.

Longitudinal Zernike coefficients describing corneal aberrations were computed for each measurement using least-squares fitting up to the 6th polynomial order. Comparisons between lens designs were also based on paired inter-eye differences using the same signed-rank approach. Temporal plots for right and left eyes were presented for visualisation only and do not imply statistical independence between fellow eyes.

## 3. Results

This study evaluated the effects of spherical and aspherical Ortho-K lens designs on ocular structure and optics over 12 months. A total of 48 participants (96 eyes) had complete 12-month follow-up data and were included in the analysis. Baseline demographic and refractive characteristics were comparable between lens-design eyes.

The analysis focused on longitudinal changes in corneal shape, optical power distribution, and ocular aberrations derived from Zernike polynomial fitting. Both lens designs produced the expected corneal reshaping pattern associated with Ortho-K: central flattening surrounded by peripheral steepening. Yet, differences emerged in the magnitude and stability of these effects.

Figure 2 illustrates the longitudinal changes in tangential corneal power observed in a representative Ortho-K subject over 12 clinical visits. The maps depict the difference in tangential power relative to baseline, with warmer colours indicating areas of increased curvature and cooler tones showing flattening. The central corneal region (A_1_) progressively flattens following lens wear, while a distinct peripheral steepening zone (A_2_) emerges and stabilises over time. Both areas were automatically identified using a custom-built MATLAB code, which segmented the flattened and steepened zones based on topographic curvature patterns. This spatial progression reflects the expected corneal remodelling associated with Ortho-K treatment, characterised by a central reduction in refractive power averaged as P_1_, surrounded by a compensatory peripheral increase, averaged as P_2_.

**Figure 4:**
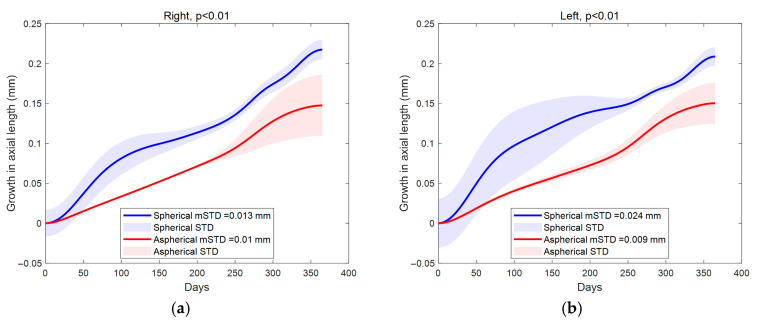

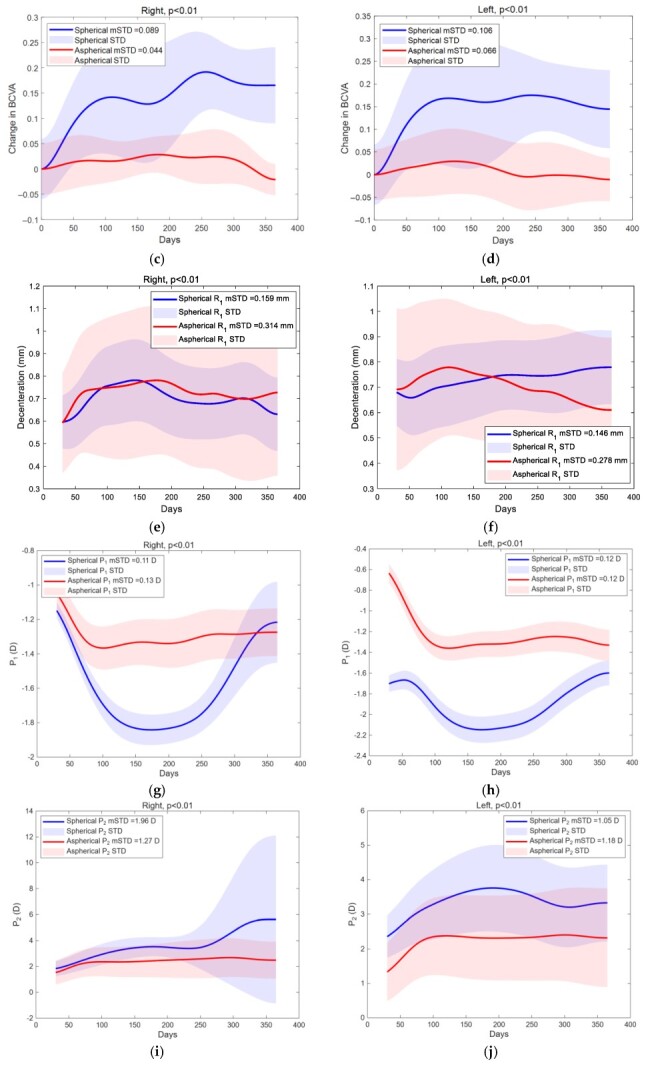

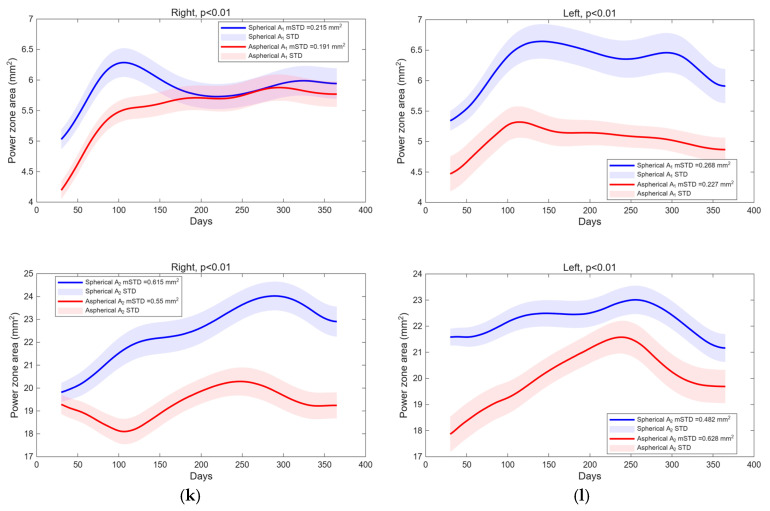
Comparison of ocular changes over time between spherical and aspherical lens designs in right and left eyes. (**a**,**b**) Axial length (AL) growth; (**c**,**d**) changes in best-corrected visual acuity (BCVA) in decimal units; (**e**,**f**) lens decentration; (**g**,**h**) first-order optical power (P_1_); (**i**,**j**) second-order optical power (P_2_); (**k**,**l**) power area measurements (A_1_ and A_2_). Data are plotted over 365 days, with statistically significant differences observed across the plotted parameters.

As shown in Figure 4, the subplots illustrate longitudinal changes in key ocular parameters following lens wear with either a spherical or an aspherical design over 12 months. Subplots (a) and (b) show that AL increased in both eyes over time, with significantly greater elongation observed in the spherical lens group. Similarly, BCVA, shown in subplots (c) and (d), exhibited greater fluctuation with spherical lenses, particularly during the early phase of treatment. The mean baseline BCVA was 0.86 ± 0.25 in spherical-lens eyes and 0.98 ± 0.25 in aspherical-lens eyes, expressed in decimal units.

Lens decentration differed between designs and was statistically significant in both eyes (e–f, *p* < 0.01), despite overlapping standard deviation bands. Optical power profiles revealed notable distinctions: P1 was consistently more negative with spherical lenses (g–h), while P_2_ remained higher in this group (i–j). Power area measurements (k–l) further supported these differences, with spherical lenses producing larger treatment zones (A_1_ and A_2_) throughout the follow-up period. These findings suggest that lens geometry influences axial growth, visual function, and optical quality over time.

The monthly axial elongation rate was calculated from the average change in AL over the 12 months (*m* = 12 in Equation (6)), expressed as a percentage of the baseline value and divided by the number of months:

(6) $$Monthly\;growth\;rate\left(\%\right)=\left(\frac{{AL}_{m}-{AL}_{0}}{{m\;AL}_{0}}\right)\times 100$$

where $AL_{0}$ and $AL_{m}$ represent the mean ALs at baseline and after m months, respectively. Although axial elongation is more commonly reported as an absolute change in millimetres, the present percentage-based calculation was used as a supplementary descriptive index to normalise growth relative to the initial eye size of each lens-design group. This was intended to allow proportional comparison of axial growth between spherical and aspherical lens conditions, while absolute AL change in millimetres remained the primary clinically interpretable outcome. The reported values (0.21% for spherical and 0.10% for aspherical lenses) were obtained from a combined analysis of both eyes across all participants, as each subject contributed one eye per lens design in the contralateral-eye study setup.

As shown in Figure 4, AL increased in both lens groups over 12 months, but the increase was greater in eyes fitted with spherical lenses. By month 12, axial elongation was approximately 0.21~0.22 mm in spherical-lens eyes and 0.15 mm in aspherical-lens eyes, corresponding to an inter-design difference of roughly 0.06~0.07 mm. Changes in BCVA were also larger with spherical lenses, particularly during the early phase of treatment, whereas the aspherical design remained close to baseline by 12 months. Lens decentration differed significantly between designs in both eyes (*p* < 0.01). Corneal power measurements showed that P_1_ was generally more negative with spherical lenses, while P_2_ remained higher in the spherical group, indicating stronger peripheral steepening. The treatment zones were also larger with spherical lenses: at 12 months, A_2_ was approximately 22.9 vs. 19.3 mm^2^ in right eyes and 21.2 vs. 19.7 mm^2^ in left eyes for spherical versus aspherical designs, respectively. Overall, these data indicate that lens geometry influenced axial elongation, corneal power redistribution, and treatment-zone behaviour over time.

**Figure 5:**
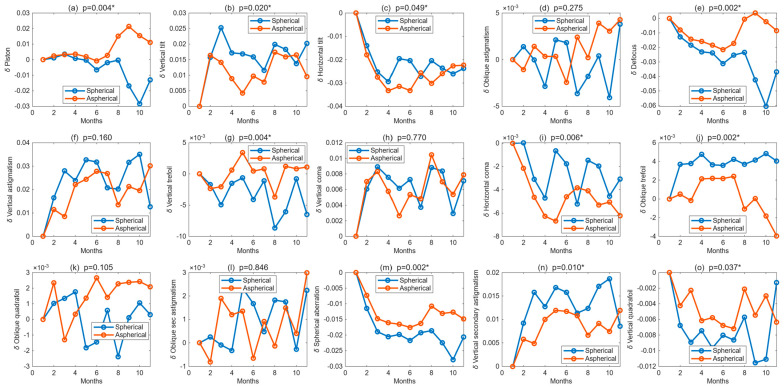
Longitudinal changes in Zernike polynomial coefficients for right eyes fitted with spherical and aspherical Ortho-K lenses over 12 months. Subplots show, from left to right and top to bottom: (**a**) piston, (**b**) vertical tilt, (**c**) horizontal tilt, (**d**) oblique astigmatism, (**e**) defocus, (**f**) vertical astigmatism, (**g**) vertical trefoil, (**h**) vertical coma, (**i**) horizontal coma, (**j**) oblique trefoil, (**k**) oblique quadrifoil, (**l**) oblique secondary astigmatism, (**m**) spherical aberration, (**n**) vertical secondary astigmatism, and (**o**) vertical quadrifoil. *p*-values indicate the statistical significance of between-design comparisons, where * indicates significance.

Figure 5 shows the longitudinal evolution of Zernike-derived corneal aberration coefficients in right eyes fitted with spherical and aspherical lenses over 12 months. The clearest between-design differences were observed for vertical tilt (*p* = 0.02), vertical coma (*p* = 0.006), and spherical aberration (*p* = 0.002), all of which were lower and less variable in the aspherical group than in the spherical group. Additional significant differences were observed in other Zernike terms associated with treatment-zone asymmetry and optical stability (*p* < 0.05), whereas oblique astigmatism and vertical trefoil did not differ significantly between designs (*p* > 0.1). Overall, the right-eye data indicate that the aspherical design produced a more stable higher-order aberration profile over time.

**Figure 6:**
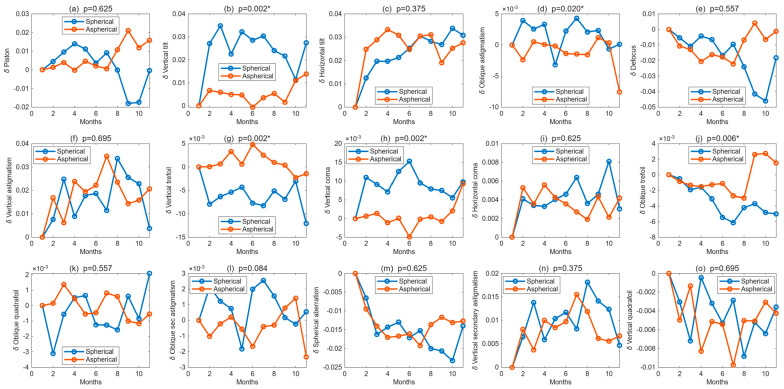
Longitudinal changes in Zernike polynomial coefficients for left eyes fitted with spherical and aspherical Ortho-K lenses over 12 months. Subplots show, from left to right and top to bottom: (**a**) piston, (**b**) vertical tilt, (**c**) horizontal tilt, (**d**) oblique astigmatism, (**e**) defocus, (**f**) vertical astigmatism, (**g**) vertical trefoil, (**h**) vertical coma, (**i**) horizontal coma, (**j**) oblique trefoil, (**k**) oblique quadrifoil, (**l**) oblique secondary astigmatism, (**m**) spherical aberration, (**n**) vertical secondary astigmatism, and (**o**) vertical quadrifoil. *p*-values indicate the statistical significance of between-design comparisons, where * indicates significance.

A similar pattern was observed in the left eyes (Figure 6). Compared with the spherical design, the aspherical lens again showed lower and more stable aberration coefficients, with significant between-design differences for vertical tilt (*p* = 0.004), horizontal tilt (*p* = 0.002), vertical coma (*p* = 0.027), and oblique trefoil (*p* = 0.002). In contrast, oblique astigmatism and vertical quadrifoil did not differ significantly between lens designs (*p* > 0.05). Taken together, the bilateral Zernike results support the view that the aspherical BOZ geometry yielded a more stable corneal optical profile than the spherical design.

## 4. Discussion

Myopia has risen sharply over recent decades, especially in many Asian populations and most notably among young people [22,23,24,25]. This trend increases the likelihood of progressing to high myopia, a condition associated with severe ocular complications [26,27]. Ortho-K lenses are one of the most effective interventions for slowing myopic progression [28,29], but not all designs perform equally well [30,31]. This study, therefore, compared two Ortho-K lens designs not only to determine which is more effective but also to identify the reasons for their differing performance.

Over 12 months, the aspherical Ortho-K lens consistently produced a more stable corneal optical profile than the spherical design, with smaller fluctuations in key higher-order aberrations, steadier treatment-zone behaviour, and corneal reshaping patterns consistent with a more stable peripheral myopic-defocus profile. This combination offers a coherent explanation for why the aspherical geometry performed better.

A widely accepted explanation for the myopia-control effect of Ortho-K is that it acts, at least in part, by imposing a relative peripheral myopic defocus on the retina [32,33,34]. Meta-analyses and broad reviews confirm meaningful reductions in axial elongation with Ortho-K compared with controls, thereby situating our findings within a well-established therapeutic effect [12]. Focused reviews further emphasise peripheral defocus as a plausible biological signal, although the exact amount required remains debated [35]. This interpretation aligns with recent clinical evidence showing that aspheric base-curve Ortho-K lenses produce distinct corneal reshaping profiles and greater myopia-control efficacy compared with spherical designs [36].

Design details matter because they shape the on-eye optics that drive this signal. Independent clinical work shows that smaller treatment zones and, within limits, modest decentration are associated with slower axial growth in children wearing Ortho-K. In other words, concentrating the mid-peripheral steepening strengthens the retinal cue without unduly sacrificing central image quality [14]. This is consistent with contemporary summaries and updates on Ortho-K efficacy [37]. The optical patterns observed in the aspherical lens group, with more compact and stable power profiles, align well with this mechanism. Previous work confirms that back-optic-zone diameter and lens geometry significantly influence corneal power redistribution and treatment-zone formation [38].

More specifically, our findings are consistent with previous studies showing that treatment-zone characteristics are closely related to axial elongation in orthokeratology. Lin et al. [14] reported that smaller treatment zones, particularly when accompanied by modest decentration, were associated with slower axial growth. Similarly, Guo et al. [39] showed that lenses with a smaller back optic zone diameter produced a smaller treatment zone and were associated with less axial elongation. Zhang et al. [40] further demonstrated that lens geometry influences corneal power redistribution and that these redistribution patterns are associated with subsequent axial elongation. Our results fit well within this framework: the aspherical design produced a more compact and stable treatment zone, together with slower axial elongation, suggesting that its geometric modification may improve myopia-control efficacy by shaping a more favourable corneal power profile. Li et al. [41] reported a similar relationship between aspheric lens-induced corneal power redistribution and reduced axial elongation, which further supports the interpretation of the present findings.

There is also a plausible aberrational explanation. While Ortho-K inevitably alters higher-order aberrations, the clinical objective is to achieve a predictable profile that preserves daytime vision while maintaining the peripheral cue. Evidence suggests that aspheric base-curve designs can modulate spherical aberration and coma differently from conventional spherical designs, sometimes increasing controlled spherical aberration without degrading subjective visual performance, provided the treatment zone remains well-behaved. The current study’s data showed less variability in vertical coma and spherical aberration with the aspherical lens, indicating better centration and stability, as well as fewer unwanted optical effects. This interpretation mirrors findings that aspheric base-curve Ortho-K can deliver favourable HOA profiles while maintaining visual quality [42]. The maintained visual quality in the aspherical design may be attributed to the novel combined design, which keeps the central 4 mm zone spherical, ensuring clear central optics. In contrast, the peripheral aspheric zone shapes a stable treatment profile. Consistent with this, Li et al. [41] found that aspheric Ortho-K lenses modify corneal power distribution and reduce axial elongation more effectively than spherical lenses, supporting the optical mechanism proposed here.

The broader literature reports that Ortho-K induces peripheral myopic defocus across eccentricities in children and adolescents, reinforcing the biological plausibility behind design-driven differences such as those observed here [34]. Taken together, these findings suggest that the aspherical geometry may outperform the spherical one by tightening the treatment zone, stabilising centration, and producing corneal reshaping patterns that are more consistent with a stable peripheral defocus profile, thereby improving the optical environment associated with slower axial growth [14].

This study has several limitations. First, although the data were derived from a two-centre study conducted under a shared protocol, the completed sample comprised 48 paired participants. This was sufficient to detect moderate inter-design differences, but smaller effects, particularly for secondary optical outcomes, may have gone undetected, and the findings may not fully generalise to other clinical settings, patient populations, and fitting practices. Second, central and peripheral refraction were not measured directly but were inferred from corneal power redistribution and aberration patterns; accordingly, interpretations regarding peripheral myopic defocus should be regarded as indirect and hypothesis-supporting rather than directly demonstrated. Third, the follow-up period was limited to 12 months, which captures short- to medium-term treatment effects but does not establish whether the observed differences in axial elongation and optical stability would persist over longer periods of myopia control. These limitations should be considered when interpreting the present findings.

Nevertheless, the overall pattern observed here is consistent with previous studies suggesting that aspheric lens geometry and treatment-zone behaviour may contribute to improved optical stability and reduced axial elongation in Ortho-K [18,40,43].

The consistently sharper optics achieved with the aspherical design are due to its geometry, which promotes a more compact, stable treatment zone. This configuration minimises fluctuations in key higher-order aberrations, especially spherical aberration and coma, thereby enhancing overall optical quality. The smaller and steadier treatment zone maintains a concentrated refractive reshaping area, thereby supporting an optical profile consistent with a more stable peripheral myopic-defocus pattern, which may contribute to axial growth regulation [14,35,39].

In addition, the aspherical lens design maintains a corneal reshaping pattern consistent with this peripheral defocus cue without compromising central image clarity, enabling effective myopia control while preserving daytime vision quality. Although lens decentration differed significantly between designs in both eyes (Figure 4e,f, *p* < 0.01), the aspherical design still showed lower variability in key higher-order aberrations and a more stable treatment-zone profile.

## 5. Conclusions

In this retrospective analysis of data from a previously conducted two-centre contralateral-eye clinical study, aspherical Ortho-K lenses were associated with slower axial elongation and a more stable corneal optical profile than spherical lenses over 12 months. Compared with the spherical design, the aspherical lens produced a more compact and stable treatment zone and lower variability in key higher-order aberrations. These findings suggest that localised aspheric modification of the back optic zone may improve myopia-control performance while maintaining optical stability. Further multicentre studies with longer follow-up and direct peripheral refraction measurements are needed to confirm these effects.

## Figures and Tables

**Table 1:** Baseline corneal surface and power measurements represented in terms of the arithmetic average (mean), variation around the mean as a standard deviation (STD) and the most common values (Mode).

	Flat Sim-K (D)	Steep Sim-K (D)	AL (mm)	HVID (mm)	Sph (D)	Cyl (D)	Axis (°)
Mean	42.76	44.14	24.45	11.30	−2.09	−0.40	75.54
STD	±1.28	±1.33	±0.78	±0.32	±1.08	±0.49	±83.21
Mode	42.26	44.88	24.53	11.13	−1.25	0	0

## Data Availability

The datasets generated and analysed in the current study are available from the corresponding author on reasonable request. Not all data are publicly available due to considerations regarding potential future commercialisation.

## Abbreviations

The following abbreviations are used in this manuscript:

AL	axial length
BCVA	best-corrected visual acuity
BOZ	back optic zone
Dk	oxygen permeability
IOP	intraocular pressure
Ortho-K	orthokeratology
RC	reverse curve
RGP	rigid gas-permeable
RMSE	root mean square error
VST	vision shaping treatment
